# β-hydroxybutyrate impairs monocyte function *via* the ROS-NLR family pyrin domain-containing three inflammasome (NLRP3) pathway in ketotic cows

**DOI:** 10.3389/fvets.2022.925900

**Published:** 2022-08-29

**Authors:** Zhihao Dong, Xudong Sun, Yan Tang, Shengbin Luo, Hongdou Jia, Qiushi Xu, Qianming Jiang, Juan J. Loor, Wei Xu, Chuang Xu

**Affiliations:** ^1^Heilongjiang Provincial Key Laboratory of Prevention and Control of Bovine Diseases, College of Animal Science and Veterinary Medicine, Heilongjiang Bayi Agricultural University, Daqing, China; ^2^Mammalian NutriPhysioGenomics, Department of Animal Sciences and Division of Nutritional Sciences, University of Illinois, Urbana, IL, United States; ^3^Department of Biosystems, Biosystems Technology Cluster, KULeuven, Geel, Belgium

**Keywords:** ketosis, monocytes, β-hydroxybutyric acid, oxidative stress, phagocytosis

## Abstract

Cows with ketosis display severe metabolic stress and immune dysfunction which renders them more susceptible to infections. Monocytes, one of the major subtypes of white blood cells, play an important role in innate immune defense against infections. Thus, the aim of this study was to investigate alterations in immune function, reactive oxygen species (ROS) production and activity of the NLR family pyrin domain containing 3 (NLRP3) inflammasome pathway in monocytes (CD14^+^) of cows with clinical ketosis (CK). Twelve healthy multiparous Holstein cows [blood β-hydroxybutyrate (BHB) concentration < 1.2 m*M*] and 12 cows with CK (BHB > 3.0 m*M*) at 3 to 14 days in milk were used for blood sample collection. To determine effects of BHB on phagocytosis, ROS and protein abundance of the NLRP3 inflammasome pathway *in vitro*, monocytes isolated from healthy cows were treated with 3.0 m*M* BHB for 0, 6, 12 or 24 h. Dry matter intake (22.7 vs. 19.0 kg) was lower in cows with CK. Serum concentrations of fatty acids (0.30 vs. 0.88 m*M*) and BHB (0.52 vs. 3.78 m*M*) were greater in cows with CK, whereas concentration of glucose was lower (4.09 vs. 2.23 m*M*). The adhesion, migration and phagocytosis of monocytes were lower in cows with CK, but apoptosis and ROS content were greater. Protein abundance of NLRP3, cysteinyl aspartate specific proteinase 1 (caspase 1) and interleukin-1B p17 (IL1B p17) were greater in monocytes of cows with CK, while abundance of NADPH oxidase isoform 2 (NOX2) was lower. Compared with 0 h BHB, ROS content and apoptosis were greater in the monocytes challenged for 6, 12 or 24 h BHB. Compared with 0 h BHB, protein abundance of NLRP3, caspase 1, IL1B p17 and concentration of IL1B in medium were greater in the monocytes challenged for 6, 12 or 24 h BHB. However, compared with 0 h BHB, protein abundance of NOX2 and phagocytosis of monocytes were lower in the monocytes challenged for 6, 12 or 24 h BHB. Overall, the data suggested that exogenous BHB activated the ROS-NLRP3 pathway, which might be partly responsible for immune dysfunction of dairy cows with CK.

## Introduction

Ketosis is a common metabolic disease that occurs with greatest frequency during the transition period. This disease is a severe risk factor for infectious disorders such as metritis and mastitis (1). It has been demonstrated that dairy cows with ketosis had increased odds (from 1.0 to 5.8) of developing metritis after calving (2). A large portion of this response is due to severe metabolic stress and immune dysfunction (3, 4). For instance, chemotactic capacity of leukocytes was lower in ketotic cows (5). In addition, a large number of studies reported that increased circulating concentrations of ketone bodies [β-hydroxybutyrate (BHB), acetoacetic acid and acetone] impaired function of immune cells (6–8). Monocytes, components of immune cells of the innate system, have emerged as important regulators of infectious disorders. It is unknown to what extent immune function of monocytes is affected by ketosis and whether BHB plays a direct role.

Oxidative stress is a major contributing factor of immune dysfunction, which increases the susceptibility of transition dairy cows to infectious diseases (9). An imbalance between generation of reactive oxygen species (ROS) and the system's ability to neutralize and eliminate them causes oxidative stress. Elevated circulating concentrations of fatty acids and BHB underscore that a major contributing factor for the increase in ROS in ketotic cow is the severe metabolic stress (10–12). Work with human monocytes revealed that overproduction of ROS enhanced apoptosis and hampered immune function (13).

The ROS-sensitive NLR family pyrin domain containing 3 (NLRP3) inflammasome is an innate immune sensor, which provides an immediate response against pathogen invasion. The activation of the NLRP3 inflammasome requires a priming step, typically by a TLR ligand [such as LPS (14)] that leads to upregulation of transcription of the inflammasome components along with inflammatory genes. Besides TLR ligands, in non-ruminants, activation of the NLRP3 inflammasome triggered by ROS leads to oligomerization of the protein with adaptor molecule apoptosis-associated speck-like protein containing a CARD (ASC) (15, 16). Following auto-activation through inflammasome assembly, cysteinyl aspartate specific proteinase 1 (caspase 1) cleaves interleukin-1B (IL1B), which mediates the immune response of monocytes (17, 18). It is now well-established that dairy cows with ketosis display systemic oxidative stress with elevated concentrations of IL1B in the blood and impaired immune function (19, 20). Given the negative associations between immune function and metabolic stress, we hypothesized that BHB may activate the ROS-NLRP3 inflammasome in monocytes and contribute to immune dysfunction during ketosis. The aim of this study was to investigate (1) immune function of monocytes in ketotic cows and (2) the effects of BHB on the ROS-NLRP3 inflammasome as well as immune function of monocytes *in vitro*.

## Materials and methods

### Animals

The experiment protocol was approved by the Ethics Committee on the Use and Care of Animals at Heilongjiang Bayi Agricultural University (Daqing, China) approved the study protocol (Number of permits: SY201912007). In the present study, Holstein cows with similar number of lactations (median = 3, range = 2 to 4) and days in milk (DIM: median = 8 d, range = 3 to 14 d) were selected from a 2,000-cow dairy farm in Daqing, Heilongjiang, China. Experimental cows underwent routine physical examinations to guarantee the absence of other comorbidities. Cows had *ad libitum* access to a TMR consisting of 4.5% hay (alfalfa hay and oat hay), 49.3% corn silage, and 46.2% concentrate and mineral mix on a DM basis (DM: 46.4 ± 1.3%), with free access to tap water. Individual cow feed intake was recorded by farm staff, and DM percentage of the TMR was used to calculate daily DMI. If a nitroprusside test for ketone bodies in milk was positive and in the presence of clinical symptoms including excessively dry feces, rapid loss of live weight, decreased feed intake and milk yield, the cows were classified as suspected clinical ketosis (CK) (2, 21, 22). According to clinical symptoms and serum BHB concentrations (22–24), 12 CK cows with whole blood BHB concentrations >3.0 m*M* and 12 healthy cows (without clinical symptoms) with whole blood BHB concentrations below 1.2 m*M* were selected.

Blood samples were harvested by coccygeal venipuncture and then centrifuged at 2,000 × g for 10 min at 4°C to obtain serum. Whole blood concentrations of BHB in these cows were measured with an electrochemical blood ketone meter (TNN-II, Yicheng Biotechnology Co. Ltd., Beijing, China). Serum concentrations of glucose (F006-1-1, Jiancheng, Nanjing, China) and fatty acids (A042-1-1, Jiancheng) were determined using an autoanalyzer (Hitachi 7170, Tokyo, Japan) with commercially-available kits. The basic description of the cows used is reported in Table 1.

**Table 1:** Basic description of Holstein cows classified as healthy control (*n* = 12) and clinical ketosis (CK, *n* = 12).

	**Healthy (*****n*** = **12)**		**Clinical ketosis (*****n*** = **12)**		
**Item**	**Median**	**Interquartile range**	**Median**	**Interquartile range**	***P*-value**
Body weight (kg)	613	588–639	625	601–648	*P* = 0.540
DMI (kg)	22.7	20.3–24.5	19.0	18.1–20.2	*P* = 0.004
BHB (m*M*)	0.52	0.18–0.83	3.78	3.55–4.08	*P* < 0.001
Serum fatty acids (m*M*)	0.30	0.20–0.41	0.88	0.68–1.06	*P* = 0.002
Serum glucose (m*M*)	4.09	3.86–4.32	2.23	2.07–2.37	*P* < 0.001

### Isolation of blood mononuclear cells

Density gradient separation was used to isolate mononuclear cells from blood. Holstein cows were bled by coccygeal venipuncture and blood were collected in 20 mL Sodium heparin anticoagulation tube (Junnuo, Shandong, China). Samples were then diluted in an equal amount of phosphate buffer saline (PBS) containing 0.02% ethylene diamine tetraacetic acid (EDTA), layered on Biocoll Separating Solution before centrifuging at 800 × g for 30 min. The interphase containing mononuclear cells was collected according to manufacturer's instructions (P5280, Solarbio, Beijing, China) and shaken for 40 s to lyse erythrocytes (R1010, Solarbio) (250 x g for 10 min). Then, mononuclear cells were washed twice with PBS containing 5 g/L of bovine serum albumin (BSA) and 2.0 m*M* EDTA (250 × g for 10 min).

### Magnetic activated cell sorting of CD14^+^ monocytes, cell culture and treatment

Cells were suspended in PBS containing 5 g/L of BSA and 2 m*M* EDTA (MACS-buffer, 130-091-221, Miltenyi Biotec, Bergisch Gladbach, Germany), and cell aggregates were removed using Pre-Separation Filters (130-042-201, Miltenyi Biotec). Viable mononuclear cells were counted and incubated with anti-bovine CD14 monoclonal antibody (MCA2678GA, Bio-red, California, USA) in MACS-buffer for 30 min at 4°C. Mononuclear cells were centrifuged (250 × g for 5 min) and suspended in MACS-buffer before incubating with MicroBeads (130-407-101, Miltenyi Biotec) in MACS-buffer for 15 min at 4°C. Thereafter, cells were centrifuged (250 × g for 5 min) and suspended in MACS-buffer. Monocytic separation was performed using MACS MS (middle size) columns according to the manufacturer's instructions. To verify the purity of CD14^+^ monocytes isolated from blood mononuclear cells, the expression of CD14 was detected by immunofluorescence (FLUOVIEW FV1000 microscope, Olympus, Tokyo, Japan).

In experiment 1, CD14^+^ monocytes were isolated from healthy and CK cows for subsequent experiments. For the ROS content and phagocytosis analyses, dead cells were excluded by addition of propidium iodide (25). In experiment 2, isolated CD14^+^ monocytes from healthy cows were seeded into 6-well plates with Iscove's modified Dulbecco's medium (IMDM, include 4.0 m*M* L-Glutamin; SH30228.01B, Hyclone, Los Angeles, USA) supplemented with 10% fetal bovine serum (FBS; FB15015, Clark, Cordova, Argentina), 100 U/mL Penicillin/Streptomycin. Cells were incubated at 37°C and 5% CO_2_ for 24 h (25, 26). The CD14^+^ monocyte was cultured with 3.0 m*M* BHB (H6501, Sigma-Aldrich, St Louis, USA) for 0, 6, 12 or 24 h. The BHB was solubilized in ultrapure sterile water filtered through a 0.22-μm-pore-size filter. For ROS content and phagocytosis analyses, dead cells were excluded by addition of propidium iodide (25).

### Adhesion assay

The adhesion assay of CD14^+^ monocytes was performed according to a previously described method (27). Briefly, 24 well plates were precoated with 200 μL FBS for 2 h. A total of 10^5^ monocytes (200 μL) were isolated from healthy and CK cows and incubated at 37°C with 5% CO_2_ for 1 h. Monocytes were then fixed with fixative (4% paraformaldehyde, P1110, Solarbio) for 15 min at room temperature. Unbound cells were removed by washing with PBS. Monocytes were stained with Hoechst 33342 (C0021, Solarbio). and then observed by fluorescence microscopy (Tis, Nikon, Sendai-shi, Japan). At least 3 vision fields were counted in each of 3 wells per group. Area counting was analyzed with the ImageJ software (version 1.8.0, National Institutes of Health, Bethesda, MD) for each group.

### Migration assay

For transwell migration assays, 100 μL cell suspension (1 × 10^5^ cells) were placed in the top chamber of the transwell system (353097, Corning, NY, USA) with serum-free medium. Six-hundred μL medium with 10% FBS were added to the lower chamber of the transwell system. The top chambers were removed after incubating at 37 °C for 1 h (27). Migratory cells in the lower chambers were collected and measured by flow cytometry (Sysmex Cyflow Cube8, Sysmex). Subsequently, results were analyzed with Flowjo (TreeStar, Ashland, OR). To exclude debris, CD14^+^ monocytes were gated based on forward and side scatter (FSC/SSC) parameters. The migration was analyzed by the number of monocytes.

### Apoptosis assay

Apoptosis of CD14^+^ monocytes was measured *via* the annexin V-FITC/propidium iodide (PI) apoptosis detection kit (C1062M, Beyotime, Shanghai, China). In brief, after treatment as indicated above, cells were washed and centrifuged (300 × g for 5 min) with PBS, and 1× Binding Buffer (300 μL) was used to resuspend CD14^+^ monocytes. Then, CD14^+^ monocytes were stained with Annexin V-FITC (10 μL) for 15 min, followed by staining with 5 μL PI for 5 min at room temperature. After mixing with 1× Binding Buffer (200 μL), cells were analyzed by flow cytometry (Sysmex Cyflow Cube8, Sysmex). Flow cytometric compensation was established using a single stain control for each fluorochrome and unstained cells. To exclude debris, CD14^+^ monocytes were gated based on forward and side scatter (FSC/SSC) parameters. Data acquisition was completed when exactly 10,000 events were acquired in the region of counting beads. The cellular apoptosis (Annexin-V-FITC+) was analyzed by calculating the fluorescence intensity.

### Measurement of ROS

Intracellular ROS content was detected using a commercial ROS assay kit (S0033S, Beyotime Institute of Biotechnology, Jiangsu, China) according to protocols from the supplier. After treatment as indicated above, CD14^+^ monocytes were incubated with 20 μ*M* 2′,7'-dichloro-fluorescein diacetate for 30 min at 37 °C. Cells were then washed twice and centrifuged (300 × g for 5 min) with PBS. After resuspending with serum-free medium, cells were measured by flow cytometry (Sysmex Cyflow Cube8, Sysmex) and analyzed with Flowjo. To exclude debris, CD14^+^ monocytes were gated based on forward and side scatter (FSC/SSC) parameters. PI^−^ was selected to remove dead cells. Data acquisition was completed when exactly 10,000 events were acquired in the region of counting beads. The ROS production was expressed as the mean fluorescence intensity (MFI) calculated using Flowjo software.

### Bacteria and phagocytosis

*S. aureus* bacteria labeled with fluorescein isothiocyanate (FITC) (College of Life Sciences, Heilongjiang Bayi Agricultural University, China) was grown at 37°C in Soybean-Casein Digest Agar medium with 25 μg/mL erythromycin overnight. After culturing to the mid-exponential phase (OD600 = 0.8–0.9), bacteria were washed 3 times and resuspended with PBS containing 0.2% BSA. After treatment as indicated above, CD14^+^ monocytes were infected with live *S. aureus* with green fluorescence (10 *S. aureus* with green fluorescence per cell were added) at 37 °C in a humidified atmosphere of 5% CO_2_ for 1 h (27, 28). Monocytic phagocytosis was then terminated in an ice box and cells washed with PBS. Phagocytosis assay of CD14^+^ monocyte was measured by flow cytometry (Sysmex Cyflow Cube8, Sysmex) and analyzed with Flowjo (27). Unstimulated controls were used to determine gating strategy for flow cytometry. To exclude debris, CD14^+^ monocytes were gated based on forward and side scatter (FSC/SSC) parameters. PI^−^ was selected to remove dead cells. Data acquisition was completed when exactly 10,000 events were acquired in the region of counting beads. Then, phagocytosis was analyzed through calculating the fluorescence intensity (after subtracting the negative control).

### Immunofluorescence assay

After, CD14^+^ monocytes were washed 3 times with PBS before fixed with fixative (4% paraformaldehyde, P1110, Solarbio) at room temperature for 20 min. The cell membrane was stained with Dil fluorescent cell membrane marker for 20 min (5m*M*, abs42002237, Absin, Shanghai, China) after washing 3 times with PBS. CD14^+^ monocytes were stained by 4′,6-diamidino-2-phenylindole (DAPI, 10 μg/mL, D8417, Sigma) for 10 min. Lastly, cells were washed 3 times in PBS and imaged with an Olympus FLUOVIEW FV1000 microscope (Olympus).

### Protein extraction and western blotting

Protein abundance was measured using protocols described previously by our group (29). Total protein was extracted from CD14^+^ monocytes with a commercial protein extraction kit (99% RIPA cell lysis buffer and 1% phosphatase inhibitors, R0010 and P0100, Solarbio) following the supplier's instructions. Protein concentration was measured with the bicinchoninic acid protein assay kit (P0012, Beyotime), and protein samples (15 μg per lane) separated using 10% SDS-PAGE with known molecular weight markers (P1200, Solarbio). Subsequently, the protein was transferred onto polyvinylidene difluoride (PVDF) membranes and blocked in Tris-buffered saline/Tween (TBST) supplemented with 3% BSA for 4 h. Membranes were then incubated with primary antibodies against NADPH oxidase isoform 2 (NOX2, 1:1000, ab129068, Abcam, Cambridge, MA), NLRP3 (1: 2000, ab214185, ABclonal, Wuhan, China), caspase 1 (1:1000, 22915-1-AP, Proteintech Group, Inc, Chicago, USA), IL1B (1:500, 66737-1-lg, Proteintech), and glyceraldehyde-3-phosphate dehydrogenase (GAPDH; 1:2000, abs132004, Absin) at 4°C overnight. Membranes were then washed with TBST and incubated with secondary antibodies conjugating with horseradish peroxidase (respective anti-mouse or anti-rabbit, BA1038 or BA1039, Boster, Wuhan, China) at room temperature for 45 min. Lastly, immunoreactive bands were visualized by protein imager (AI600, GE Healthcare, Marlborough, MA; UVP, Analytikjena German) through an enhanced chemiluminescence solution (abs920, Absin). ImageJ analysis software (NIH, Bethesda) was used to quantify the intensity of bands.

### ELISA

The concentrations of IL1B were assessed with a commercially-available ELISA kit (SEA563Bo, USCN Life Science Inc., Wuhan, China) according to the manufacturer's instructions. The minimum detectable concentration was 6.5 pg/mL. This assay was run in triplicate for each sample, and absorbance values read at 450 nm using a spectrophotometer (51119100; Thermo Fisher Scientific, Shanghai, China).

### Cell viability

Cell viability was assessed using a Cell Counting Kit 8 (Cck8; C0037, Biyuntian Co., Shanghai, China) according to the manufacturer's instructions. The cells (10^4^) were allowed to grow in a 96-well plate. Afterward 10 μL of Cck8 was added to each well, and the culture medium incubated at 37°C for an additional 2 h. Absorbance values were read at 450 nm using a spectrophotometer (51119100; Thermo Fisher Scientific). Cell viability was calculated as a percentage of the Control cells.

### Statistical analysis

Data analyses were performed using GraphPad Prism program (Prism 7, GraphPad InStat Software). Each experiment was repeated at least three times. The Shapiro-Wilk and Levene tests were performed to analyze normality and homogeneity of variance for all data. In the *in vivo* studies, the data of baseline characteristics were not normally distributed and were analyzed with the Wilcoxon signed-rank test. Other data were normally distributed and analyzed with paired *t*-tests. In the *in vitro* studies, data were normally distributed and analyzed with one-way ANOVA followed by a Bonferroni correction. Linear and quadratic contrasts were conducted to evaluate time-dependent effects of BHB. Data of baseline characteristics were expressed as the median and interquartile range (IQR), and other data reported as means ± standard error of the means (mean ± SEM). *P* < 0.05 was considered statistically significant.

## Results

### Content of fatty acids, BHB, and glucose in serum of cows with CK

Body weight did not differ between cows with CK and healthy cows (*P* = 0.540, Table 1). However, DMI of cows with CK was lower (*P* = 0.004, Table 1). Concentrations of fatty acids (*P* = 0.002) and BHB (*P* < 0.001) of cows with CK were greater (Table 1). In contrast, serum concentration of glucose was lower in cows with CK (*P* < 0.001, Table 1).

### Immune function in monocytes of cows with CK

**Figure 1:**
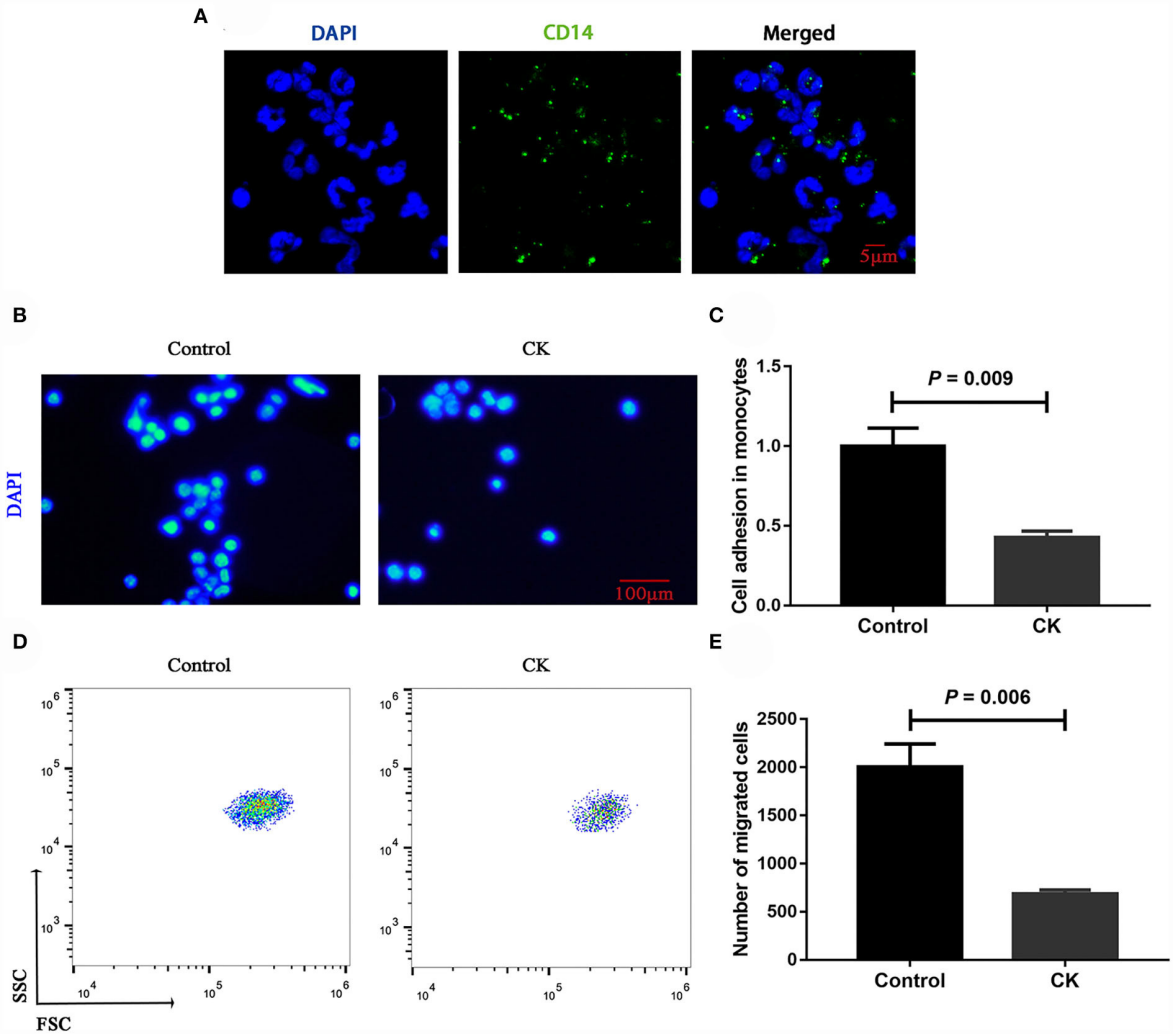
Migration and adhesion of CD14^+^ monocytes in healthy cows (*n* = 12), and clinically ketotic cows (CK, *n* = 12). **(A)** Representative images of CD14 (green), and nuclei (blue), scale bar = 5 μm. **(B,C)** Adhesion of monocytes, Nuclei (blue), scale bar = 100 μm. **(D,E)** Migration of monocytes. Data were analyzed with paired *t*-tests and reported as means ± SEM of three independent experiments.

**Figure 2:**
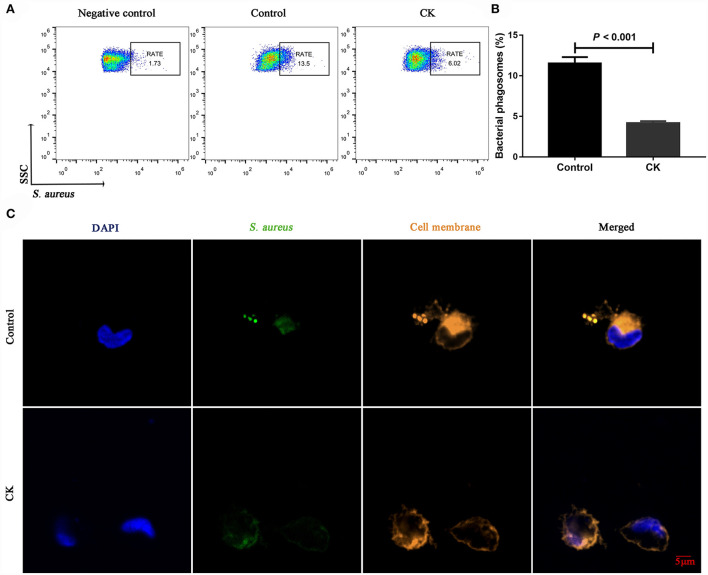
Phagocytosis of CD14^+^ monocytes in healthy cows (*n* = 12), and clinically ketotic cows (CK, *n* = 12). **(A,B)** Phagocytosis of monocytes, RATE, proportion of positive cells in 10,000 cells. **(C)** Representative images of *Staphylococcus aureus* (green) and cell membrane (orange), scale bar = 5 μm. Data were analyzed with paired *t*-tests and reported as means ± SEM of three independent experiments.

Immunofluorescence staining results revealed successful purification of CD14^+^ monocytes from mononuclear cells (Figure 1A). Monocytes of cows with CK had lower CD14^+^ adhesion (*P* = 0.009, Figures 1B,C) and migration (*P* = 0.006, Figures 1D,E) along with lower phagocytosis (*P* < 0.001, Figures 2A,B). Similarly, immunofluorescence staining results revealed that the phagotrophic *S. aureus* with green puncta was lower in monocytes of cows with CK (Figure 2C).

### ROS content and apoptosis in monocytes of cows with CK

**Figure 3:**
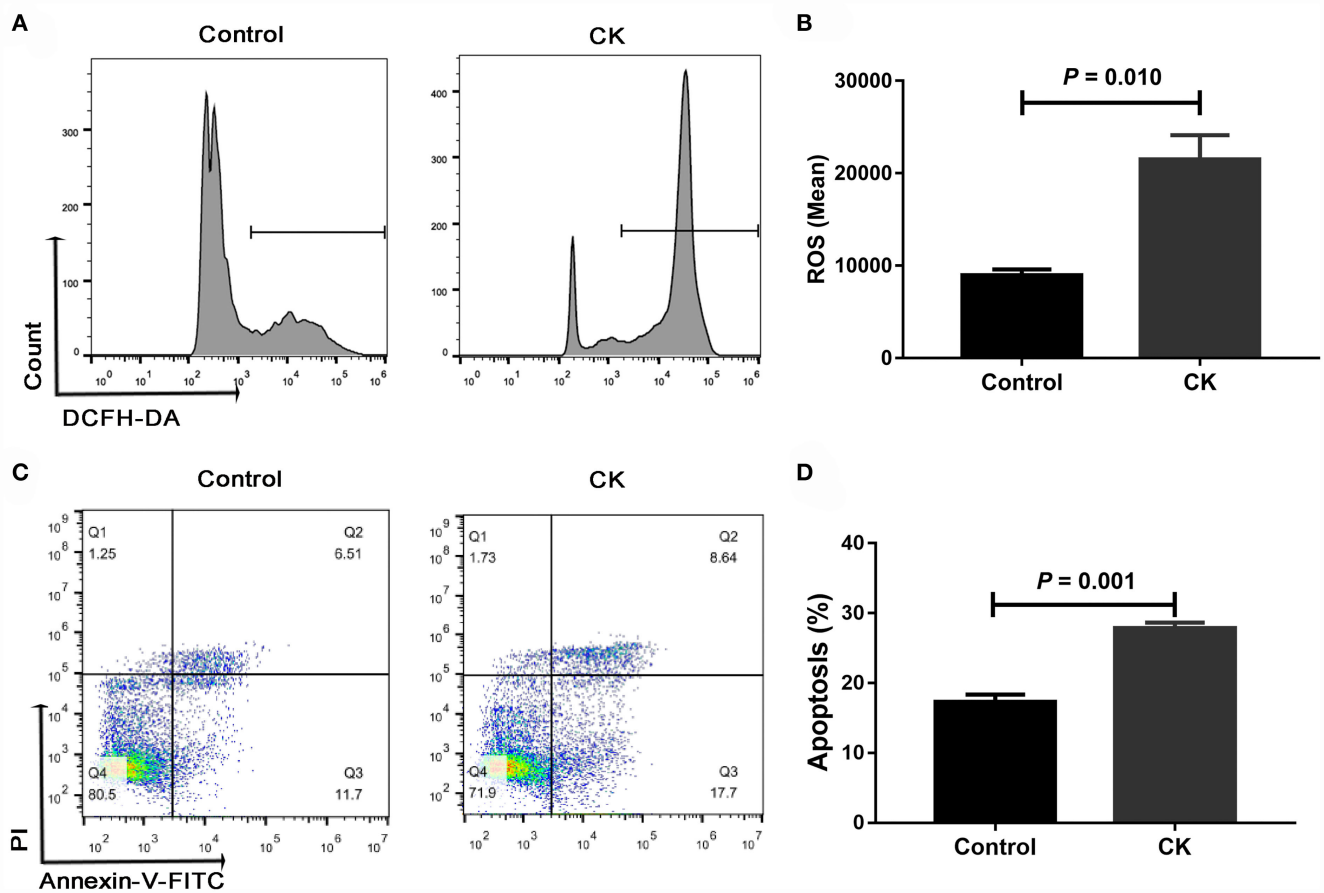
Reactive oxygen species (ROS) content and apoptosis of CD14^+^ monocytes from healthy cows (*n* = 12), and clinically ketotic cows (CK, *n* = 12). **(A,B)** ROS content in monocytes. **(C,D)** Apoptosis of monocytes. Data were analyzed with paired *t*-tests and reported as means ± SEM of three independent experiments.

Compared with healthy cows, the ROS content was greater in monocytes of cows with CK (*P* = 0.010, Figures 3A,B). Similarly, the cellular apoptosis was greater in monocytes of cows with CK compared with healthy cows (*P* = 0.001, Figures 3C,D).

### NLRP3 pathway in monocytes of cows with CK

**Figure 4:**
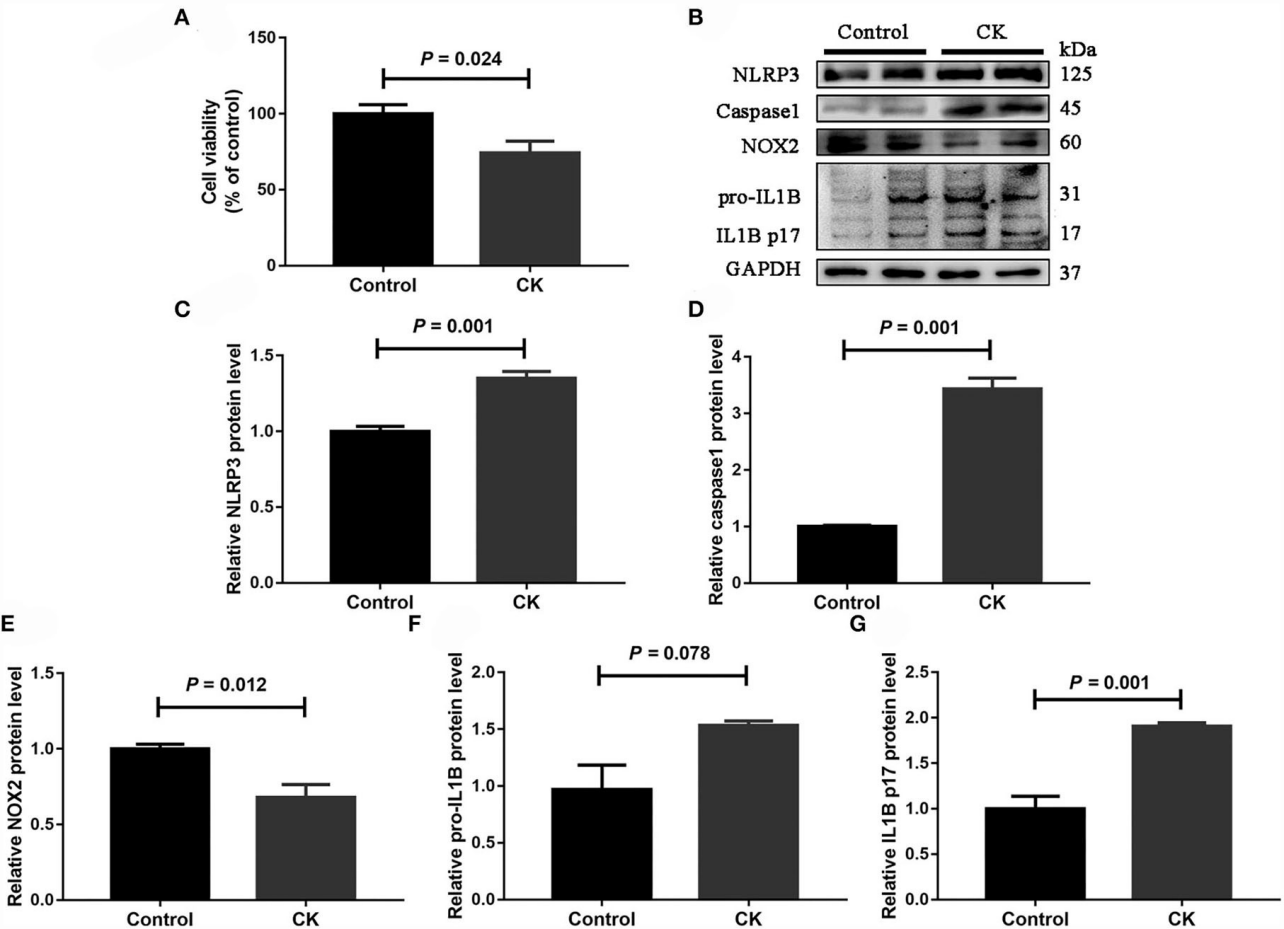
Abundance of molecules involved in the NLRP3 inflammasome pathway in CD14^+^ monocytes from healthy cows (*n* = 12), and clinically ketotic cows (CK, *n* = 12). **(A)** Monocyte cell viability. **(B)** Western blot analysis of NLRP3, caspase 1, NOX2, pro-IL1B and IL1B p17. **(C)** Protein abundance of NLRP3. **(D)** Protein abundance of caspase 1. **(E)** Protein abundance of NOX2. **(F)** Protein abundance of pro-IL1B. **(G)** Protein abundance of IL1B p17. Data were analyzed with paired *t*-tests and reported as means ± SEM of three independent experiments.

Compared with healthy cows, cell viability was lower in monocytes from cows with CK (*P* = 0.024, Figure 4A). Protein abundance of NLRP3 (*P* = 0.001, Figures 4B,C), caspase 1 (*P* = 0.001, Figures 4B,D), pro-IL1B (*P* = 0.078, Figures 4B,F) and IL1B p17 (*P* = 0.001, Figures 4B,G) was greater in monocytes of cows with CK. In contrast, protein abundance of NOX2 (*P* = 0.012, Figures 4B,E) was lower in monocytes of cows with CK.

### Effects of BHB on ROS content, apoptosis and phagocytosis in bovine monocytes

**Figure 5:**
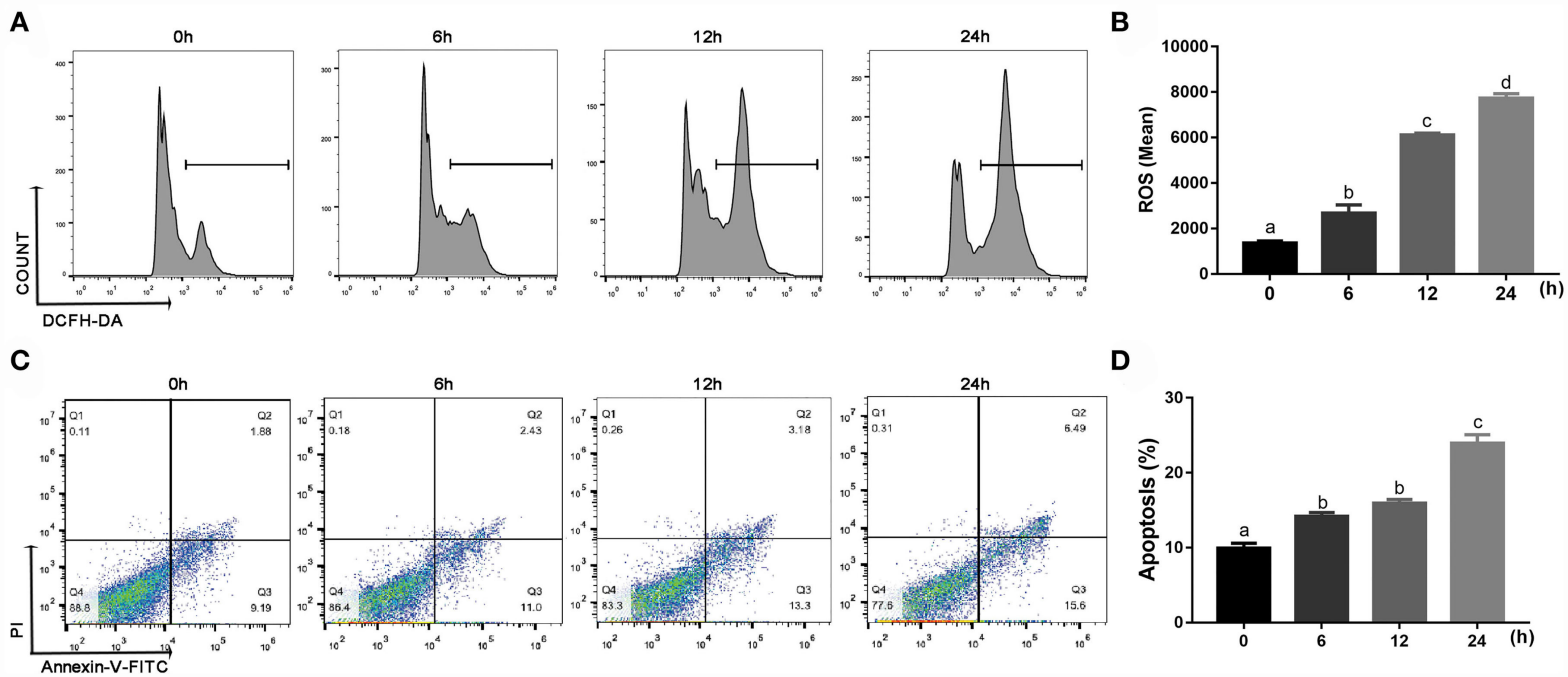
Effects of β-Hydroxybutyrate (BHB) on reactive oxygen species (ROS) content and apoptosis of CD14^+^ monocytes. Monocytes were treated with 3.0 m*M* of BHB for 0, 6, 12 or 24 h. **(A,B)** ROS content in monocytes. **(C,D)** Apoptosis of monocytes. Comparisons among groups were calculated using a one-way ANOVA with subsequent Bonferroni correction. Data are means ± SEM of three independent experiments. Different lowercase letters in the bar chart indicate significant differences (*P* < 0.05).

**Figure 6:**
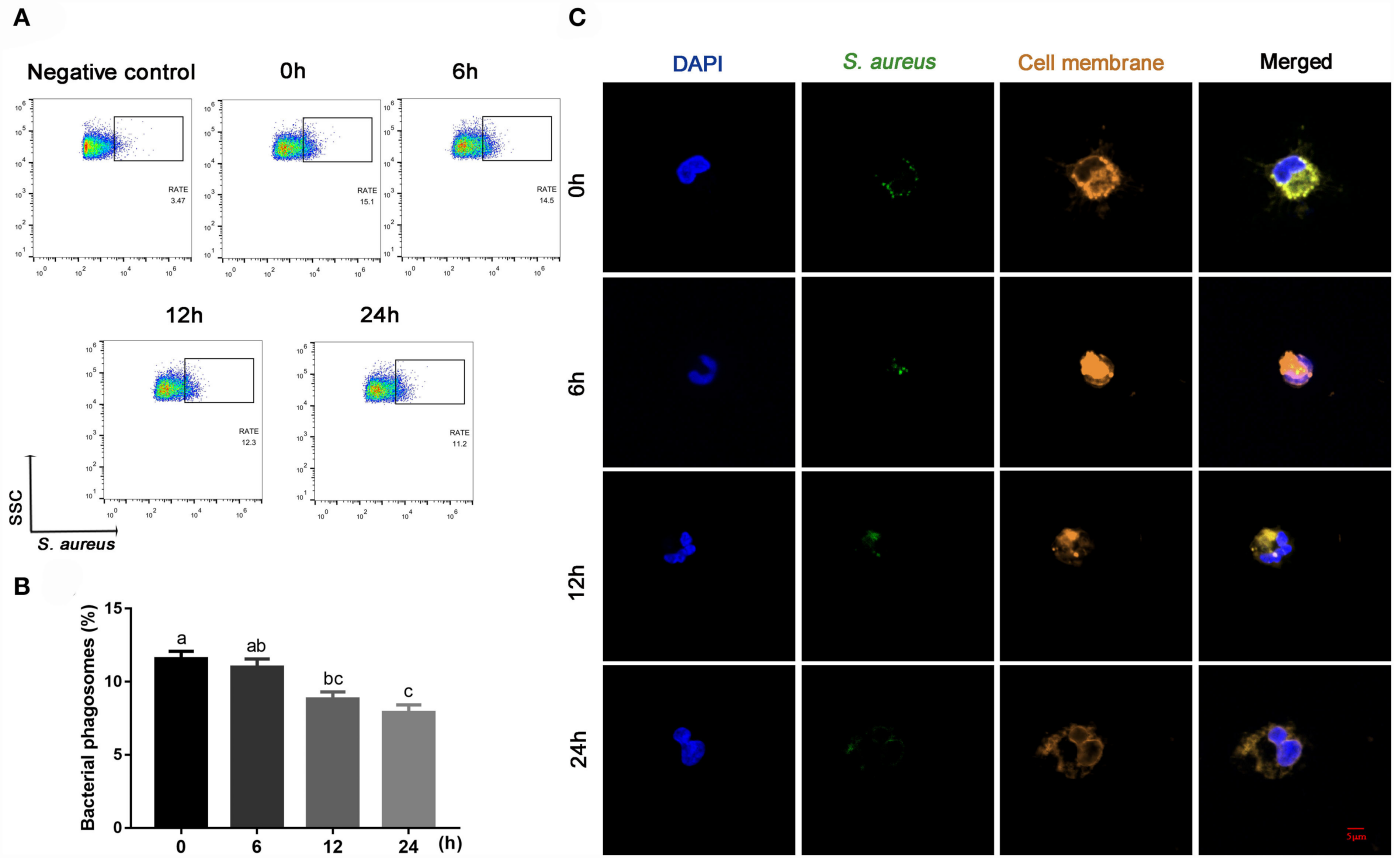
Effects of β-Hydroxybutyrate (BHB) on phagocytosis of CD14^+^ monocytes. The monocytes were treated with 3.0 m*M* of BHB for 0, 6, 12 or 24 h, and then infected with live green-fluorescence-labeled *Staphylococcus aureus* (10 *S. aureus* with green fluorescence per cell) for 1 h. **(A,B)** Phagocytosis of monocytes, RATE, proportion of positive cells in 10,000 cells. **(C)** Representative images of *Staphylococcus aureus* (green) and cell membrane (orange), scale bar = 5 μm. Comparisons among groups were calculated using a one-way ANOVA with subsequent Bonferroni correction. Data are means ± SEM of three independent experiments. Different lowercase letters in the bar chart indicate significant differences (*P* < 0.05).

Compared with 0 h, content of ROS (linear and quadratic effect, *P* < 0.001 and *P* = 0.003, Figures 5A,B; Table 2) and cellular apoptosis (linear and quadratic effect, *P* < 0.001 and *P* = 0.612, Figures 5C,D; Table 2) were higher at 6, 12 or 24 h in cells incubated with 3.0 m*M* BHB. In contrast, phagocytosis of monocytes at 6, 12 or 24 h was lower compared with the 0 h (linear and quadratic effect, *P* < 0.001 and *P* = 0.392, Figures 6A,B; Table 2). Similarly, immunofluorescence staining results revealed that the phagotrophic *S. aureus* with green puncta of monocytes at 6, 12 or 24 h was lower compared with the 0 h (Figure 6C).

**Table 2:** Linear and quadratic contrasts in monocytes incubated with 3.0 m*M* BHB over time.

** Item**	**SEM**	***P***	
		**Linear**	**Quadratic**
ROS	309.6	<0.001	0.003
Apoptosis	1.059	<0.001	0.612
Phagocytosis	0.730	<0.001	0.392
NLRP3	0.054	<0.001	<0.001
Caspase 1	0.091	<0.001	0.053
NOX2	0.083	<0.001	0.004
Pro-IL1B	0.101	=0.002	0.001
IL1B p17	0.152	<0.001	0.621
IL1B in medium	1.675	<0.001	0.079

ROS, Reactive oxygen species; NLRP3, NLR family pyrin domain containing 3 inflammasome; Caspase 1, Cysteinyl aspartate specific proteinase 1; NOX2, NADPH oxidase isoform 2; pro-IL1B, Pro-Interleukin-1β; IL1B p17, Interleukin-1β p17; IL1B, Interleukin-1β.

### Effects of BHB on abundance of the NLRP3 pathway proteins in bovine monocytes

**Figure 7:**
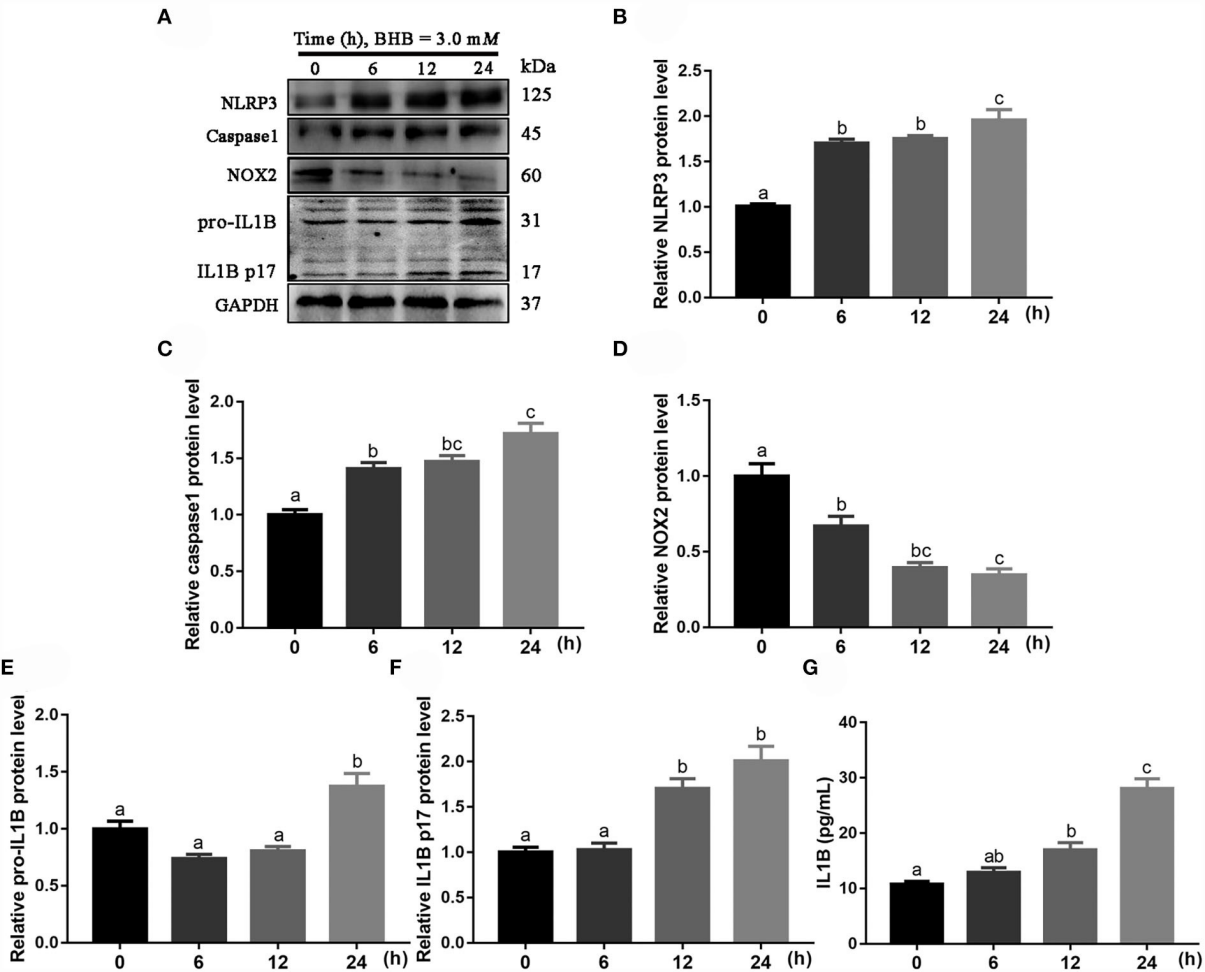
Effects of β-Hydroxybutyrate (BHB) on NLRP3 signaling pathway of CD14^+^ monocytes. The monocytes were treated with 3.0 m*M* of BHB for 0, 6, 12 or 24 h. **(A)** Western blot analysis of NLRP3, caspase 1, NOX2, pro-IL1B and IL1B p17. **(B)** Protein abundance of NLRP3. **(C)** Protein abundance of caspase 1. **(D)** Protein abundance of NOX2. **(E)** Protein abundance of pro-IL1B. **(F)** Protein abundance of IL1B p17. **(G)** Medium concentration of IL1B. Comparisons among groups were calculated using a one-way ANOVA with subsequent Bonferroni correction. Data are means ± SEM of three independent experiments. Different lowercase letters in the bar chart indicate significant differences (*P* < 0.05).

Protein abundance of NLRP3 (linear and quadratic effect, *P* < 0.001 and *P* < 0.001; Figures 7A,B; Table 2), caspase 1 (linear and quadratic effect, *P* < 0.001 and *P* = 0.053; Figures 7A,C; Table 2), pro-IL1B (linear and quadratic effect, *P* = 0.002 and *P* = 0.001; Figures 7A,E; Table 2) and IL1B p17 (linear and quadratic effect, *P* < 0.001 and *P* = 0.621; Figures 7A,F; Table 2) were greater at 6, 12 or 24 h compared with the 0 h. In contrast, protein abundance of NOX2 (linear and quadratic effect, *P* < 0.001 and *P* = 0.004; Figures 7A,D; Table 2) was lower at 6, 12 or 24 h compared with the 0 h. Compared with 0 h, the IL1B concentrations (linear and quadratic effect, *P* < 0.001 and *P* = 0.079; Figure 7G; Table 2) were greater in medium at 12 or 24 h.

## Discussion

Ketosis is a common metabolic disease during the transition period in dairy cows. Severe metabolic stress during ketosis is thought to impair immune function and increase the risk of infectious disorders, including metritis and mastitis (3, 30, 31). Work with ruminants has confirmed that leukocytes from ketotic cows and leukocytes challenged with ketone bodies have a diminished capacity for chemotaxis (8). Cows with negative energy balance (NEB) during the transition period are also characterized by increased inflammation and immune dysfunction (32). In the present study, immune function of monocytes (CD14^+^) was impaired in ketotic cows partly due to direct negative effects of high BHB concentrations. Increases in BHB lead to greater ROS production and activation of the NLRP3 inflammasome pathway. Thus, our data contributed to a better understanding of the mechanisms responsible for immune dysfunction during ketosis.

Monocytes, a class of leukocytes, are an important innate immune cell of the mononuclear phagocyte system, which participates in the body's resistance to infectious diseases (33, 34). Work in humans revealed that impaired monocyte function in peripheral blood is associated with gestational diabetes mellitus physiopathology (35). Furthermore, mice with non-alcoholic fatty liver disease displayed impaired phagocytosis capacity of macrophages (36), as well as impaired migration and adhesion of monocytes (37, 38). Thus, we speculated that lower phagocytosis, migration and adhesion of blood circulating monocytes in cows with CK partly explained the higher susceptibility to infections that this disease confers to afflicted cows (39). The fact that incubation with BHB decreased monocyte phagocytosis was consistent with previous findings in bovine leukocytes (8). Overall, it is evident that increased metabolic stress impairs the immune function of monocytes in cows with CK.

Oxidative stress, an imbalance between the production of ROS and their removal, plays a crucial role in the dysfunction of immune cells, thus, contributing to the development of infectious diseases (9, 40, 41). Negative associations between the level of ROS and immune function are often observed in dairy cows with NEB during the transition period (32). An excessive production of ROS triggers cell apoptosis, which impairs immune function of macrophages (42, 43). Thus, we speculated that greater ROS content and apoptosis in monocytes from cows with CK decreases cell numbers and contributes to immune dysfunction. Metabolic challenges of dairy cows with CK are thought to be the main source of ROS. In fact, increased circulating ketone bodies (BHB and acetoacetate) were reported to enhance production of ROS in bovine hepatocytes (10, 44). In agreement with those data, in the present study, incubating exogenous BHB increased ROS content and apoptosis in monocytes, which further confirmed our data *in vivo*. As such, that effect can partly explain the observation that increases in circulating BHB in postpartal dairy cows coincides with the period in which they are most-susceptible to infectious diseases (45).

Due to the leucine-rich repeats found in the C-terminus of NLRP3, it was hypothesized to act as a cytosolic receptor and directly bind to a ligand (such as LPS) (46, 47). In addition, the NLRP3 inflammasome is a redox-sensitive inflammatory pathway that in non-ruminants regulates immune function of cells (48–50). Subsequently, caspase 1 can negatively regulate the NOX2 complex through hydrolysis of gp91 from its subunits (51), which is responsible for regulating immune function of cells. Work in non-ruminants has demonstrated that inhibition of NOX2 decreased phagocytosis of macrophages (52), and increased the risk of infection in patients (53).

In the present study, the greater protein abundance of NLRP3, caspase 1 and IL1B along with lower protein abundance of NOX2 underscored the mechanistic association between BHB and the NLRP3-NOX2 pathway. In fact, there is a positive association between circulating BHB concentrations and activity of the NLRP3 inflammasome in liver and mammary gland tissue of ketotic cows (54, 55). It is noteworthy, however, that some studies have reported that BHB inhibits of the NLRP3 inflammasome, e.g., in mouse macrophages (56) or neutrophils (57). The discrepancy in the response of the NLRP3 inflammasome between published data and the present study may be due to the fact that in previous non-ruminant studies BHB increased antioxidant capacity [murine nerve cells (58); HEK293 cells, (59)], whereas in bovine hepatocytes (10) and the present study ROS content was increased in response to BHB. Oral ketone supplementation acutely increased markers of NLRP3 inflammasome activation in human monocytes (60). Similarly, psychological stress associated with increased blood BHB levels was correlated with activation of NLRP3 in the prefrontal cortex (61). Thus, taken together, previous studies are indicative that BHB can activate the NLRP3 inflammasome in the absence of ligands, which agrees with our data.

In general, along with the upregulation of the NLRP3-NOX2 pathway, the downregulation of phagocytosis in monocytes cultured with BHB suggested that BHB inhibited phagocytosis of monocytes *via* activating the NLRP3 inflammasome pathway. Together with our data *in vivo*, we speculate that increased metabolic stress may inhibit phagocytosis of monocytes *via* overactivation of NLRP3 inflammasomes.

## Conclusions

Monocytes of ketotic cows display an activation of the ROS-NLRP3 inflammasome pathway as a direct effect of high concentrations of BHB. Overactivation of the ROS-NLRP3 inflammasome decreased phagocytosis, indicating this pathway is responsive to BHB and can cause immune dysfunction. Overall, our data contribute to increasing the understanding of mechanisms responsible for immune dysfunction during ketosis.

## Data availability statement

The raw data supporting the conclusions of this article will be made available by the authors, without undue reservation.

## Ethics statement

The experiment protocol was approved by the Ethics Committee on the Use and Care of Animals at Heilongjiang Bayi Agricultural University (Daqing, China). Written informed consent was obtained from the owners for the participation of their animals in this study.

## Author contributions

ZD, CX, and XS conceived the study. ZD, YT, SL, HJ, QX, JL, WX, and XS carried out experiments and data analysis. XS, QJ, JL, and ZD interpreted the data. ZD, JL, and XS wrote the manuscript. All authors approved the final version.

## Funding

This work was supported by the National Natural Science Foundation of China (Beijing, China, Grant Nos. 32002348 and 32072931), Heilongjiang Touyan Innovation Team Program, the Earmarked Fund for CARS36, and the Natural Science Foundation of Heilongjiang Province Joint Guidance Project (LH2020C085).

## Conflict of interest

The authors declare that the research was conducted in the absence of any commercial or financial relationships that could be construed as a potential conflict of interest.

## Publisher's note

All claims expressed in this article are solely those of the authors and do not necessarily represent those of their affiliated organizations, or those of the publisher, the editors and the reviewers. Any product that may be evaluated in this article, or claim that may be made by its manufacturer, is not guaranteed or endorsed by the publisher.
